# Use of skeletal muscle index as a predictor of short-term mortality in patients with acute-on-chronic liver failure

**DOI:** 10.1038/s41598-021-92087-1

**Published:** 2021-06-15

**Authors:** Tongzeng Li, Manman Xu, Ming Kong, Wenyan Song, Zhongping Duan, Yu Chen

**Affiliations:** 1 Department of Infectious Disease, Beijing You’an Hospital Affiliated to Capital Medical University, Beijing, 100069 China; 2 Fourth Department of Liver Disease (Difficult & Complicated Liver Diseases and Artificial Liver Center), Beijing You’an Hospital Affiliated to Capital Medical University, Beijing, 100069 China; 3 Beijing Municipal Key Laboratory of Liver Failure and Artificial Liver Treatment Research, Beijing, 100069 China; 4 Department of Radiology, Beijing You’an Hospital Affiliated to Capital Medical University, Beijing, 100069 China

**Keywords:** Gastroenterology, Medical research, Risk factors

## Abstract

Sarcopenia is a well-recognized factor affecting the prognosis of chronic liver disease, but its impact on acute decompensation underlying chronic liver disease is unknown. This study evaluated the impact of sarcopenia on short-term mortality in patients with acute-on-chronic liver failure (ACLF). One hundred and seventy-one ACLF patients who underwent abdominal CT between 2015 and 2019 were retrospectively included in this study. Skeletal muscle index at the third lumbar vertebrae (L3-SMI) was used to diagnose sarcopenia.The ACLF patients in this study had a L3-SMI of 41.2 ± 8.3 cm^2^/m^2^ and sarcopenia was present in 95/171 (55.6%) patients. Body mass index (BMI), cirrhosis, and higher serum bilirubin were independently associated with sarcopenia. Following multivariate Cox regression analysis, cirrhosis (hazard ratio (HR) 2.758, 95%CI 1.323–5.750), serum bilirubin (HR 1.049, 95%CI 1.026–1.073), and international normalized ratio (INR) (HR 1.725, 95%CI 1.263–2.355) were associated with 3-month mortality (*P* < 0.05), whereas L3-SMI and sarcopenia were not. A subgroup analysis of the factors related to sarcopenia showed that sarcopenia was still not predictive of short-term outcome in ACLF patients. L3-SMI and sarcopenia are not associated with short-term mortality in patients with ACLF.

## Introduction

Sarcopenia is an important hallmark of malnutrition, especially in patients with liver disease complicated by synthesis and metabolism disorders^1^. In recent years, an increasing number of studies have used the skeletal muscle index at the third lumbar vertebrae (L3-SMI) to determine sarcopenia status in patients with liver disease^2–5^. L3-SMI is the muscle cross-sectional area (cm^2^) at the level of the third lumbar vertebra (L3) normalized by the square of the height (m^2^) and calculated only for patients who have undergone abdominal computed tomography (CT) or magnetic resonance imaging (MRI).

Currently, the cut-off value of L3-SMI to identify sarcopenia is inconsistent, and is highly variable between Eastern and Western countries due to different ethnicities, and liver disease etiologies. The more widely used definition of sarcopenia in patients with liver disease in Western populations comes from a multi-center cohort of patients awaiting liver transplantation, which proposed that an L3-SMI of < 50 cm^2^/m^2^ in men and < 39 cm^2^/m^2^ in women correlates best with waitlist mortality in patients with end-stage liver disease^6^. In Asia, the assessment criteria for sarcopenia in liver disease proposed by the Japanese Society of Hepatology are L3-SMI < 42 cm^2^/m^2^ for males and L3-SMI < 38 cm^2^/m^2^ for females^7^. Although the evaluation criteria for sarcopenia differ, the impact of sarcopenia on adverse outcomes in patients with liver disease has been consistently recognized.

In patients with cirrhosis awaiting liver transplantation (LT), sarcopenia has been shown to be a significant risk factor for waitlist mortality, postoperative complications, and post-LT death^8–10^. Similarly, it has been shown that sarcopenia increases 1-year mortality after surgical resection of hepatocellular carcinoma (HCC)^11^ and can predict long-term survival in patients with HCC^12^.

It is well known that ACLF has a high case fatality rate, and active exploration of indicators to determine prognosis is valuable in guiding treatment. To the best of our knowledge, there has been no study on the impact of sarcopenia on the outcomes of patients with acute-on-chronic liver failure (ACLF). This study aimed to explore the prognostic value of sarcopenia diagnosed with L3-SMI for predicting short-term mortality in ACLF.

## Materials and methods

### Patients

A retrospective cohort study of ACLF patients hospitalized between January 1, 2015 and June 30, 2019 in the Beijing You'an Hospital was conducted. Patients who met the following criteria were included: (1) 18 years of age or older; (2) underwent abdominal CT within 2 weeks of hospitalization; (3) diagnosed with ACLF according to the relevant diagnostic criteria of the Asian Pacific Association for the Study of the Liver (APASL)^13^. Patients were excluded if they had any of the following conditions: (1) complicated by HCC or other malignant tumors; (2) severe basic diseases of extrahepatic organs, such as respiratory failure or heart failure; (3) complicated by other consumptive diseases, such as tuberculosis or hyperthyroidism; (4) patients with neuromuscular diseases and those who were long-term bedridden; and (5) patients who had undergone long-term corticosteroid treatment.

All patient data were retrieved from electronic medical records. Follow-up was documented for 3 months after admission. The study procedures conformed to the ethical guidelines of the Declaration of Helsinki, and were approved by the Ethics Committees of Beijing You’an Hospital (LL-2021-018-K). As this was a retrospective study, informed consent was waived.

### Clinical data

The demographic data, laboratory examination and diagnosis and treatment information of the patients were collected, including sex, age, height, body mass, and complications, such as ascites and hepatic encephalopathy. Laboratory data were also collected during the diagnosis of ACLF, including routine blood tests, liver function (total bilirubin, albumin), renal function (creatinine), electrolytes (blood sodium) and coagulation related indices. The prognosis of the patients was followed up for 90 days (non-liver transplantation survival rate). The patients' Model End-Stage Liver Disease (MELD) scores were calculated.

ACLF patients often have body fluid retention such as edema and ascites. In this study, the dry weight of ACLF patients with body fluid retention was calculated and corrected according to the clinical severity of ascites minus a certain amount of body weight^14^ (mild 5%, moderate 10%, severe 15%, and 5% if there was peripheral edema). The body mass index (BMI) was calculated according to the following formula: BMI = dry weight (kg)/height squared (m^2^), and a BMI ≥ 24 kg/m^2^ was recognized as obesity in accordance with the BMI assessment criteria in China.

### Assessment of skeletal muscle area on CT and sarcopenia

Abdominal CT examinations were performed in all patients within 2 weeks after the day of admission. CT scanning was performed with the LightSpeed VCT CT 64 scanner, USA. Skeletal muscle area (cm^2^): the cross-sectional area (cm^2^) of skeletal muscle at the third lumbar level (L3) on CT imaging estimates human skeletal muscle content, including psoas major, vertical spinal muscle, psoas muscle, transverse abdominis muscle, abdominal internal oblique muscle, abdominal external oblique muscle and rectus abdominis. The total skeletal muscle area of the L3 cross-section was evaluated by two imaging physicians independently. When disagreement occurred, a third physician was involved and an agreement was reached.

The skeletal muscle index was calculated as the area of skeletal muscle at the L3 level divided by the square of height (m^2^) to obtain the L3-SMI^6^. L3-SMI values less than 42 cm^2^/m^2^ for males and less than 38 cm^2^/m^2^ for females were considered to show sarcopenia^7^.

### Statistical analysis

Statistical analyses were performed using IBM SPSS Statistics Version 23 (IBM, Armonk, NY, USA) and GraphPad Prism Version 8.0 (GraphPad Software, La Jolla, CA, USA). Continuous variables are presented as mean ± standard deviation (SD) in the case of parametric data distribution or median (interquartile range (IQR)) in the case of non-parametric data distribution. Categorical variables are presented as a percentage. The Student’s t-test was used for group comparisons of parametric data, while the Mann–Whitney-U test was used for non-parametric data. Group comparisons of categorical variables were performed using the χ^2^ test. The features associated with sarcopenia were investigated using logistic regression analysis. Clinical characteristics associated with mortality in ACLF patients were assessed using Cox regression analysis. The association between sarcopenia and mortality in subgroups was estimated by the Kaplan–Meier method and compared using a Log-rank test. *P*-values less than 0.05 were regarded as significant for 2-sided tests.

## Results

### Characteristics of the patients

We included 171 patients with a mean age of 44.5 (SD ± 10.8) years who had an established diagnosis of ACLF in this study, including 85.4% (146) males. Overall, the mean BMI in the study patients was 22.6 ± 4.0 kg/m^2^, with 31.0% being obese (Table 1). The most common etiology of ACLF was viral hepatitis (n = 115 (67.3%), followed by alcohol (29 (17%)). Liver cirrhosis was present in 108 / 171 (63.2%) patients.

**Table 1:** Baseline characteristics of ACLF patients with and without sarcopenia.

Variables	All patients (n = 171)	Sarcopenia (n = 95)	No sarcopenia (n = 76)	*P* value
Age, yr, SD	44.5 ± 10.8	44.7 ± 10.7	44.3 ± 11.0	0.801
Sex, male, n (%)	146 (85.4)	78 (82.1)	68 (89.5)	0.175
**BMI, kg/m**^**2**^**, SD**	22.6 ± 4.0	20.9 ± 2.9	24.8 ± 4.2	< 0.001
< 24.0 (non-obese), n (%)	118 (69.0)	81 (85.3)	37 (48.7)	< 0.001
≥ 24.0 (obese), n (%)	53 (31.0)	14 (14.7)	39 (51.3)	
Liver cirrhosis, n (%)	108 (63.2)	72 (75.8)	36(47.4)	< 0.001
**Etiology of liver disease, n (%)**				0.417
Hepatitis B virus	115 (67.3)	62 (65.3)	53 (69.7)	
Alcohol	29 (17)	18 (18.9)	11 (14.5)	
Hepatitis B virus and alcohol	16 (9.4)	7 (7.4)	9 (11.8)	
Other	11 (6.4)	8 (8.4)	3 (3.9)	
Hepatic encephalopathy, n (%)	40 (23.4)	22 (23.2)	18 (23.7)	0.936
Ascites, n (%)	133 (77.8)	78 (82.1)	55 (72.4)	0.128
MELD score, IQR	22.4 ± 6.3	21.8 ± 6.5	23.1 ± 6.1	0.208
Serum sodium, mmol/L, SD	134.5 ± 4.7	133.6 ± 4.6	135.5 ± 4.5	0.009
Serum creatinine, μmol/L, IQR	60.0 (48.0–71.7)	64.5 (53.1–76.9)	55.5 (47.2–69.7)	0.009
Serum bilirubin, mg/dL, IQR	20.9 (12.5–27.5)	23.5 (15.1–30.2)	18.3 (11.0–23.9)	0.007
Serum albumin, g/L, SD	29.9 ± 5.6	29.4 ± 5.3	30.4 ± 5.9	0.241
INR, IQR	2.4 (2.1–3.0)	2.4 (2.0–2.9)	2.3 (2.1–3.2)	0.876
Platelets, ×10^9^/L, IQR	96 (66–141.8)	55.5 (47.2–69.7)	100.5 (64.5–150.8)	0.618
L3-SMI, cm^2^/m^2^, SD	41.2 ± 8.3	35.4 ± 4.7	48.4 ± 5.9	< 0.001

The L3-SMI of patients with ACLF was 41.2 ± 8.3 cm^2^/m^2^, and sarcopenia was present in 95/171 (55.6%) ACLF patients, while sarcopenia was present in 78/95 (82.1%) male patients and 17/25 (68.0%) female patients.

### Characteristics of sarcopenia defined by L3-SMI in patients with ACLF

Of the 95 ACLF patients with sarcopenia, they frequently had liver cirrhosis (*P* < 0.001), a lower BMI (*P* < 0.001), and a lower number of patients were obese, compared with non-sarcopenic patients (Table 1). Moreover, sarcopenic patients had higher serum levels of creatinine (*P* = 0.009) and bilirubin (*P* = 0.007), and lower serum levels of sodium (*P* = 0.009) compared with non-sarcopenic patients (Table 1). However, there was no significant difference in the number of patients with ascites and hepatic encephalopathy between the sarcopenic and non-sarcopenic ACLF patients. Patient characteristics in the two groups are shown in Table 1.

Univariate regression analysis showed that lower BMI (non-obesity), liver cirrhosis, lower serum sodium and higher serum bilirubin were associated with sarcopenia (Table 2). When the above variables were included in the multivariate logistic regression model, it was found that obesity (OR, 0.159, 95%CI 0.073–0.348) was a protective factor for sarcopenia. On the contrary, liver cirrhosis (OR, 2.534, 95%CI 1.218–5.274), and higher serum bilirubin (OR, 1.051, 95%CI 1.013–1.091) were associated with increased odds of sarcopenia (*P* < 0.05).

**Table 2:** Features associated with sarcopenia by logistic regression analysis in patients with ACLF.

Variables	Univariate		Multivariate	
	OR (95% CI)	*P* value	OR (95% CI)	*P* value
Obesity (≥ 24.0 kg/m^2^)	0.164 (0.079–0.338)	< 0.001	0.159 (0.073–0.348)	< 0.001
Liver cirrhosis	3.478 (1.815–6.666)	< 0.001	2.534 (1.218–5.274)	0.013
Serum sodium, per mmol/L	0.913 (0.852–0.979)	0.011		
Serum creatinine, per μmol/L	0.992 (0.981–1.003)	0.165		
Serum bilirubin, per mg/dL	1.045 (1.012–1.079)	0.007	1.051 (1.013–1.091)	0.009

**Figure 1:**
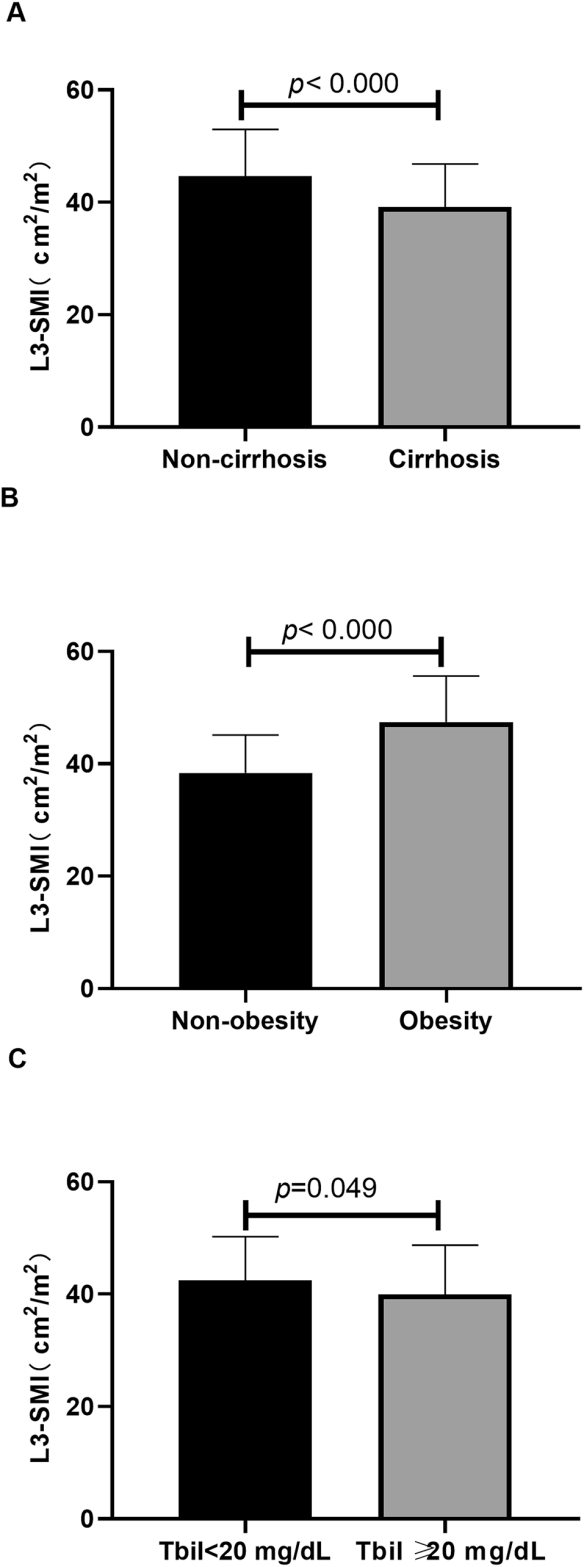
The third lumbar skeletal muscle mass index (L3-SMI) in different subgroups of ACLF patients.

The mean L3-SMI in ACLF patients with cirrhosis was 39.2 ± 7.7 cm^2^/m^2^, which was significantly lower than that in ACLF patients without cirrhosis (44.7 ± 8.3 cm^2^/m^2^) (*P* < 0.001) (Fig. 1). Mean L3-SMI was higher in obese ACLF patients compared with non-obese ACLF patients (47.4 ± 8.2 vs. 38.4 ± 6.7 cm^2^/m^2^, *P* < 0.001). Patients were divided into two groups according to the overall median serum bilirubin level: less than 20 mg/dL and greater than or equal to 20 mg/dL, and the L3-SMI in these groups was found to be 42.5 ± 7.7 cm^2^/m^2^ and 39.9 ± 8.7 cm^2^/m^2^, respectively (*P* = 0.049).

### Features associated with mortality in ACLF patients

Overall, 57 (33.3%) patients died during the 3-month follow-up period. Following univariate Cox regression analysis of patients’ clinical characteristics, liver cirrhosis (*P* = 0.014) was significantly associated with mortality in patients with ACLF. In addition, a higher MELD score (*P* < 0.001), INR (*P* < 0.001), and serum bilirubin (*P* < 0.001) and lower levels of serum sodium (*P* = 0.026) were significantly associated with mortality in patients with cirrhosis (Table 3). In addition, etiology, precipitating events did not significantly affect the short-term prognosis of ACLF (Supplementary Table 1).

**Table 3:** Features associated with mortality by Cox regression analysis in patients with ACLF.

Variables	Univariate		Multivariate	
	HR (95% CI)	*P* value	HR (95% CI)	*P* value
Age, per yr	1.022 (0.998–1.046)	0.076		
Male sex	0.897 (0.425–1.895)	0.776		
Obesity (≥ 24.0 kg/m^2^)	0.784 (0.435–1.415)	0.420		
Liver cirrhosis	2.175 (1.171–4.039)	0.014	2.758 (1.323–5.750)	0.007
Hepatic encephalopathy	0.809 (0.611–1.069)	0.136		
Ascites	1.933 (0.915–4.082)	0.084		
MELD score	1.104 (1.054–1.157)	< 0.001		
Serum sodium, per mmol/L	0.943 (0.895–0.993)	0.026		
Serum creatinine, per μmol/L	1.003 (0.996–1.010)	0.439		
Serum bilirubin, per mg/dL	1.047 (1.026–1.069)	< 0.001	1.049 (1.026–1.073)	< 0.001
Serum albumin, per g/L	0.996 (0.951–1.042)	0.851		
INR, per unit	1.922 (1.418–2.605)	< 0.001	1.725 (1.263–2.355)	0.001
Platelet	0.996 (0.991–1.000)	0.064		
L3-SMI, per cm^2^/m^2^	1.008 (0.977–1.039)	0.630		
Sarcopenia	1.054 (0.623–1.783)	0.845		

In the multivariate Cox analysis, liver cirrhosis (hazard ratio (HR) 2.758, 95%CI 1.323–5.750), serum bilirubin (HR 1.049, 95%CI 1.026–1.073), and INR (HR 1.725, 95%CI 1.263–2.355) were independently associated with mortality (*P* < 0.05) (Table 3), whereas L3-SMI and sarcopenia were not.

We further performed subgroup analyses in patients with cirrhosis and found that the association of sarcopenia with 90- and 28 day mortality was not significant, even in ACLF patients with cirrhosis (supplementary Tables 2 and 3).

### Survival in different subgroups of ACLF patients with and without sarcopenia

**Figure 2:**
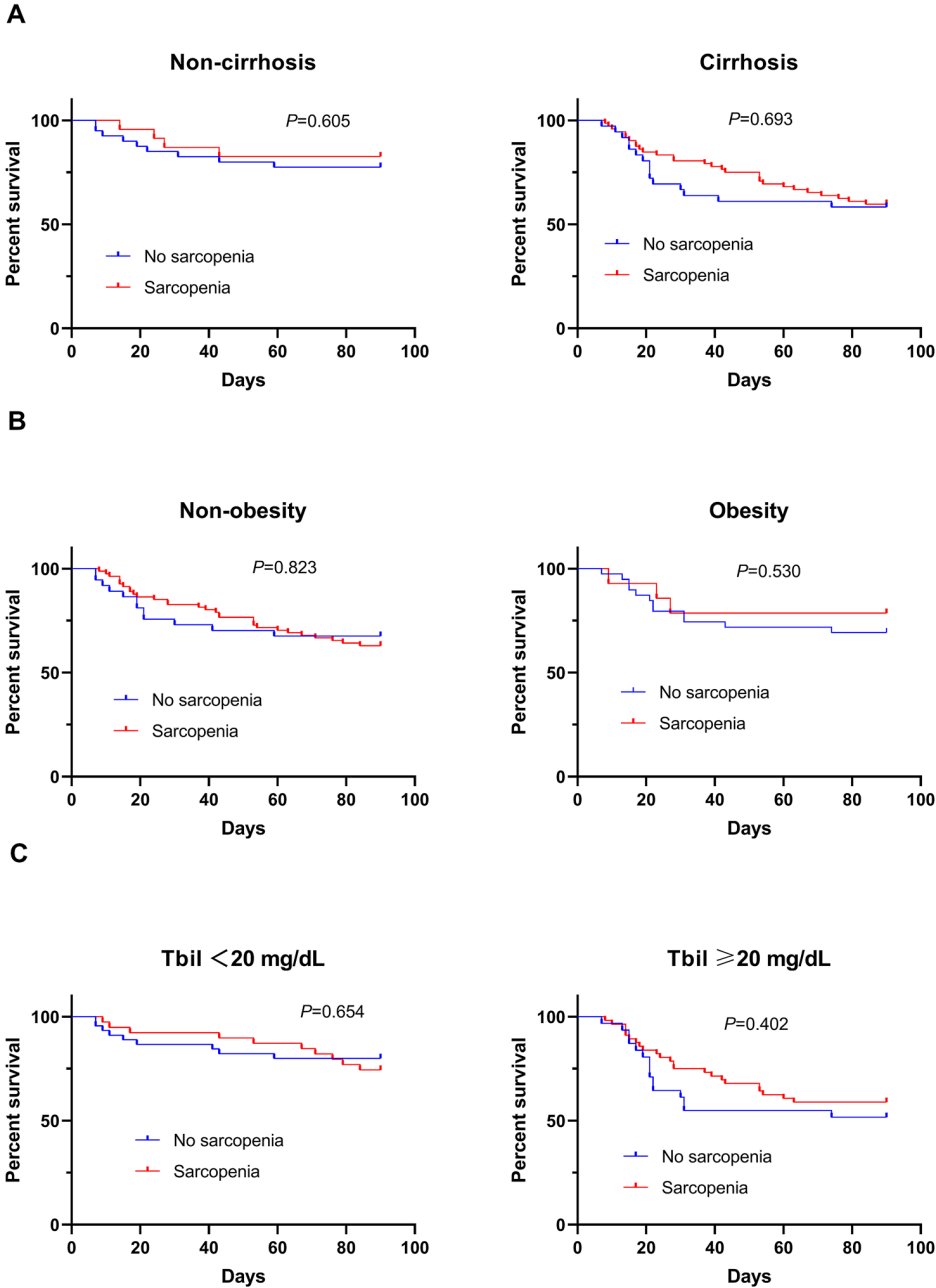
Kaplan–Meier curves indicating the survival of patients with and without sarcopenia in different subgroups of ACLF patients.

Mortality at 3 months after admission in ACLF patients with and without sarcopenia was 34.7% and 31.6%, respectively (*P* > 0.05). There was no significant difference between the cumulative survival of sarcopenic and non-sarcopenic patients in different strata of ACLF patients (stratified by the presence and absence of cirrhosis, obesity, and level of serum bilirubin) (all *P* > 0.05 by log-rank tests) (Fig. 2).

## Discussion

This is the first study to assess the impact of sarcopenia on ACLF patients. This study showed that lower BMI, liver cirrhosis, and higher serum bilirubin were associated with a higher risk of sarcopenia. However, sarcopenia does not predict short-term mortality in patients with ACLF.

Previous studies^15,16^ have shown that L3-SMI is positively correlated with BMI and that cirrhotic patients have an increased probability of developing sarcopenia than non-cirrhotic patients. The results of a study in patients with chronic HCV infection showed that skeletal muscle mass is not affected by chronic HCV infection in patients without cirrhosis, but decreases in accordance with liver disease progression^17^. The mechanisms underlying reduced skeletal muscle mass have been extensively studied in patients with liver cirrhosis. On the one hand, the gastrointestinal symptoms accompanying portal hypertension in cirrhotic patients with reduced food intake, reduced gastric reserve and impaired gastric motility, as well as malabsorption of macronutrients, reduced circulating branched chain amino acids and reduced testosterone in males can all lead to decreased skeletal muscle mass production^18,19^. On the other hand, cirrhotic patients have increased muscle breakdown due to reduced hepatic glycogen stores, resulting in increased lipid utilization and protein catabolism, with a risk of muscle breakdown even during short fasting periods such as overnight^20^. Decreased skeletal muscle mass production and increased breakdown ultimately lead to decreased muscle mass in patients with cirrhosis, which is in turn complicated by sarcopenia. The reason why high bilirubin levels are associated with low skeletal muscle mass in ACLF patients remains unclear. We speculate that patients with high bilirubin levels have a longer disease course accompanied by skeletal muscle wasting, and the specific underlying mechanism requires further study.

We also demonstrated that L3-SMI was not able to predict 3-month mortality in patients with ACLF, consistent with a previous retrospective study^21^, showing that sarcopenia is a risk factor for mortality in patients with compensated advanced chronic liver disease (CLD), but not in decompensated advanced CLD. ACLF is an acute liver decompensation that occurs on the basis of CLD, usually within 4 weeks of onset, but with mild skeletal muscle mass over a short period of time^22^, and the L3-SMI level at baseline is mainly related to the underlying liver disease basis. This study suggests that the effect on short-term mortality in patients with ACLF is on the underlying liver disease basis rather than on the L3-SMI. The potential reason for the lower performance of L3-SMI in predicting 3-month mortality in ACLF patients may be related to the evaluation of L3-SMI at a single time point without dynamic assessment. A study^23^ has shown that dynamic L3-SMI change is a predictive factor for survival in patients who underwent liver transplantation, whereas baseline L3-SMI is not. Another potential reason is that we only observed 3-month mortality in patients with ACLF, the impact of L3-SMI on longer-term mortality requires further study.

To our knowledge, the prognostic value of the skeletal muscle index for patients with liver disease is controversial. Matthew et al.^24^ found that skeletal muscle index had a limited impact on mortality in patients with end-stage liver disease awaiting liver transplantation. In addition, the findings of Amine Benmassaoud et al.^5^ suggest that sarcopenia does not worsen survival in patients with cirrhosis undergoing transjugular endoscopic portosystemic shunt for refractory ascites. Therefore, the prognostic value of L3-SMI in patients with liver disease requires further investigation.

In addition to its retrospective design, there are some limitations in this study. The present study population consisted of a low proportion of females and did not dynamically evaluate the changes in SMI during the course of ACLF with a short observation time. Additional basic research and larger clinical studies are necessary to clarify these issues.

## Conclusions

To summarize, the L3-SMI and the previously proposed definition of sarcopenia were not found to be associated with the 3-month mortality of patients with ACLF in our study cohort. The cut-off value of the skeletal muscle index for the diagnosis of sarcopenia and its impact on the prognosis of ACLF still require further study for confirmation.

## Supplementary Information

Supplementary Information.
