# Supplementary material for: Use of skeletal muscle index as a predictor of short-term mortality in patients with acute-on-chronic liver failure
Source: Sci Rep. 2021 Jun 15;11:12593. doi: 10.1038/s41598-021-92087-1 (PMC8206330; doi:10.1038/s41598-021-92087-1)
Supplement: Supplementary file 1 — Supplementary Information. [file 41598_2021_92087_MOESM1_ESM.docx]

Supplementary table 1.Association between 90-day outcome and precipitating events and etiology in patients with ACLF.

| Variable | Survivors(n=114) | | Deaths(n=57) | |
| --- | --- | --- | --- | --- |
| Precipitating events, n (%) | | |  | P value |
| Reactivation of HBV | | 24（21.1） | 15（26.3） | 0.585 |
| Alcohol | | 11（9.6） | 6（10.5） |  |
| Bacterial infection | | 10（8.8） | 2（3.5） |  |
| Drugs or Poisons | | 8（7.0） | 2（3.5） |  |
| Unclear | | 61（53.5） | 32（56.1） |  |
| Etiology of liver disease, n (%) | |  |  |  |
| Hepatitis B virus | | 74(64.9) | 41(71.9) | 0.692 |
| Alcohol | | 22(19.3) | 7(12.3) |  |
| Hepatitis B virus and alcohol | | 11(9.6) | 5(8.8) |  |
| Other | | 7(6.1) | 4(7.0) |  |

Supplementary table 2:Features associated with 28-day mortality by Cox regression analysis in ACLF patients with cirrhosis

|  | Univariate | | Multivariate | |
| --- | --- | --- | --- | --- |
| Variables | HR (95% CI) | P value | HR (95% CI) | P value |
| Age, per year | 1.020(0.978-1.060) | 0.377 |  |  |
| Male sex | 0.671(0.199-2.260) | 0.519 |  |  |
| Obesity(≥24.0 kg/m^2^) | 1.380(0.568-3.360) | 0.477 |  |  |
| Hepatic encephalopathy | 0.720(0.267-1.940) | 0.515 |  |  |
| Ascites | 1.270(0.378-4.280) | 0.698 |  |  |
| MELD score | 1.130(1.060-1.210) | <0.001 |  |  |
| Serum sodium,per mmol/L | 0.980(0.901-1.070) | 0.641 |  |  |
| Serum creatinine, per μmol/L | 0.797(0.233-2.720) | 0.718 |  |  |
| Serum bilirubin, per mg/dL | 1.050(1.020-1.080) | 0.002 | 1.044(1.008-1.082) | 0.015 |
| Serum albumin, per g/L | 1.058(0.980-1.143) | 0.149 |  |  |
| INR, per unit | 2.610(1.780-3.840) | <0.001 | 2.246(1.514-3.333) | <0.001 |
| Platelet | 0.989(0.978-0.999) | 0.037 |  |  |
| L3-SMI,per cm^2^/m^2^ | 1.030(0.983-1.090) | 0.198 |  |  |
| Sarcopenia | 1.901(0.839-4.310) | 0.124 |  |  |

Supplementary table 3:Features associated with 90-day mortality by Cox regression analysis in ACLF patients with cirrhosis

|  | Univariate | | Multivariate | |
| --- | --- | --- | --- | --- |
| Variables | HR (95% CI) | P value | HR (95% CI) | P value |
| Age, per year | 1.020(0.989-1.050) | 0.228 |  |  |
| Male sex | 0.813(0.362-1.820) | 0.616 |  |  |
| Obesity(≥24.0 kg/m^2^) | 0.809(0.389-1.680) | 0.571 |  |  |
| Hepatic encephalopathy | 1.380(0.742-2.580) | 0.306 |  |  |
| Ascites | 1.550(0.612-3.940) | 0.355 |  |  |
| MELD score | 1.090(1.050-1.150) | <0.001 |  |  |
| Serum sodium,per mmol/L | 0.957(0.902-1.010) | 0.139 |  |  |
| Serum creatinine, per μmol/L | 0.894(0.408-1.960) | 0.779 |  |  |
| Serum bilirubin, per mg/dL | 1.040(1.020-1.070) | <0.001 | 1.040(1.015-1.066) | 0.002 |
| Serum albumin, per g/L | 1.007(0.956-1.060) | 0.800 |  |  |
| INR, per unit | 2.080(1.520-2.830) | <0.001 | 1.796(1.319-2.444) | <0.001 |
| Platelet | 0.995(0.989-1.000) | 0.070 |  |  |
| L3-SMI,per cm^2^/m^2^ | 1.020(0.980-1.060) | 0.357 |  |  |
| Sarcopenia | 1.133(0.607-2.114) | 0.695 |  |  |
